# Acceptability of a social prescribing program (REDE D+) for self-care and health literacy among people with type 2 diabetes mellitus—a qualitative study

**DOI:** 10.3389/fpubh.2026.1762723

**Published:** 2026-03-19

**Authors:** Dulce Oliveira, Adriana Henriques, Paulo Nogueira, Andreia Costa

**Affiliations:** 1 Nursing Research Innovation and Development Centre of Lisbon (CIDNUR), Escola Superior de Enfermagem (ESEUL), Universidade de Lisboa, Lisbon, Portugal; 2 Escola Superior de Enfermagem de Lisboa (ESEUL), Universidade de Lisboa, Lisbon, Portugal; 3 Department of Nursing, Escola Superior de Saúde da Cruz Vermelha Portuguesa de Lisboa, Lisbon, Portugal; 4 Instituto de Saúde Ambiental (ISAMB), Faculdade de Medicina, Universidade de Lisboa, Lisbon, Portugal; 5 Laboratório Associado TERRA, Faculdade de Medicina, Universidade de Lisboa, Lisbon, Portugal; 6 Biomathematics Laboratory, Faculdade, de Medicina, Universidade de Lisboa, Lisbon, Portugal

**Keywords:** acceptability, health literacy, primary care, self-care, social prescribing, T2DM

## Abstract

**Background:**

Social prescribing (SP) interventions have been increasingly developed to improve wellbeing, promote healthier behaviors, and support the management of chronic diseases such as type 2 diabetes mellitus (T2DM). This study aims to explore the REDE D+ program, a complex SP intervention designed to promote self-care and health literacy among people with T2DM from the perspective of users in the primary health care context.

**Methods:**

A qualitative study was conducted following the implementation of a non-randomized pilot cohort study (REDE D+ program), carried out in primary health care and the community. Acceptability was assessed through two focus groups involving 18 people living with T2DM who had engaged with the SP intervention. The semi-structured interviews were transcribed and analyzed using a *reflexive thematic analysis*, following the six-phase approach proposed by Braun and Clarke. Five main themes emerged from the analysis: acceptance of the intervention, benefits, barriers and challenges, facilitators, and overall satisfaction. The perceived barriers were analyzed through the lens of the COM-B model from the *Behavior Change Wheel* theory.

**Results:**

The REDE D+ program demonstrated a high level of acceptance and satisfaction among individuals living with T2DM. The main benefits were highlighted by the adoption of healthier behaviors, such as healthy eating and physical activity, improvements in the level of knowledge and awareness about the disease, and the promotion of health literacy. Participants also reported improvements in their quality of life, including better social interaction and reduced feelings of loneliness. Regarding barriers and challenges, physical capability was related to age and osteoarticular pain, as well as difficulties in understanding; reflective motivation was related to previous preconceived ideas.

**Discussion:**

The REDE D+ program suggested that it can contribute to better self-care behaviors in T2DM and support health-related decision-making by improving health literacy. The intervention has proven to be a valid and impactful approach, with strong potential for future integration into primary health care services to support the monitoring and management of individuals with T2DM.

## Background

Type 2 diabetes mellitus (T2DM) has increased the number of new cases every day around the world, fueling the growth of high morbidity and mortality rates. This trend underscores the urgent need for sustained health promotion and early prevention of complications (1–3). The World Health Organization (WHO) reiterates the importance of strengthening global targets and pathways for health policies focused on managing modifiable risk factors, promoting early diagnosis, ensuring equity of care, and providing follow-up (2). Non-medical interventions targeting the social determinants of health are emerging to improve health behaviors and support chronic-condition management, thereby promoting more equitable care (4, 5).

Social prescribing (SP), such a complex intervention, connects patients to non-clinical community resources, contributes to the development and empowerment of people's skills and confidence, promoting intersectoral collaboration among health, social, and community environments, with significant results in the prevention and self-management of chronic diseases such as T2DM (6, 7). According to the WHO, SP allows healthcare professionals to link patients with a variety of nonclinical services within the community and volunteer sectors, aiming to enhance overall health and wellbeing (8). It is characterized, typically delivered through primary-care referral to a link worker (also known as a navigator or community health worker), whose aim is to establish a bridge between clinical and community settings (9).

The REDE D+ program, as a complex SP intervention, was designed to foster sustained engagement of individuals living with T2DM, encompassing collaborative goal setting and the selection of community-based activities tailored to their needs and personal development. Evidence shows that people living with T2DM face different challenges in managing their disease daily, need support to acquire and apply practical knowledge, set realistic goals, and solve problems (10). Some self-care behaviors, such as diet, physical activity, self-monitoring of blood glucose and feet, medication management, and stress management, are expected to be developed by a person (11). The REDE D+ program brings these fundamental T2DM care measures to the community. It provides support in integrating activities into people's daily lives, responding to their needs, and enabling the development of solid and lasting self-care behaviors.

Given the complexity of its multiple components, the REDE D+ program addressed various domains, including disease-related knowledge, health literacy, wellbeing, quality of life, and empowerment in self-care behaviors (such as diet, physical activity, and self-monitoring). According to the Medical Research Council (MRC) guidelines, the effectiveness and sustainability of a complex intervention depend on being guided by a clear theoretical model that explains how its components interact to produce the intended outcomes (12, 13). From its conception to its development, the REDE D+ program was supported by two theoretical frameworks: the *Fundamentals of Care Framework* (10, 14), which ensured that different dimensions of care were considered, and the *Behavior Change Wheel* (BCW), which facilitated the activation of individuals toward behavior change by considering their capability, motivation, and opportunity. The *Fundamentals of Care Framework* highlights the importance of core aspects of person-centered care, such as effective communication, respect for rights, patient dignity, and the involvement of the person in their care process (14–17) and the importance of the context (10, 14). The multi-component SP intervention also integrated behavior change techniques from the BCW, which supported both the initiation and development of behavioral change. This structured approach enabled the intervention to be systematically designed, ensuring it addressed the selfcare behaviors determinants previously identified in the target population through a preliminary needs assessment (18–20). By adopting a holistic approach that acknowledges the person's physical, psychosocial, and relational dimensions (15, 17) the program ensures a focus on individual needs, aiming to promote wellbeing, autonomy, and empowerment of the people living with T2DM.

Throughout the 12-week intervention, continuous support was provided to facilitate the transition and integration between primary health care and community resources, with a strong emphasis on promoting self-care behaviors and enhancing health literacy competencies. From its initial design, the REDE D+ program was developed through a co-design consensus process (21) involving individuals, professionals, and community stakeholders, aiming to address challenges identified in previous SP interventions (22, 23). An earlier study noted several barriers related to poor communication between healthcare and community stakeholders, misaligned mutual expectations and expected results, the absence of feedback on the person's journey in the community, and limited data on the post-referral process (24). To address these challenges, the SP program sought to implement a few strategies, including the development of a flowchart linking all relevant parties involved and resources such as the SP passport (21) to facilitate the interactions and communication. Understanding the difficulties experienced by participants during the SP intervention is crucial for informing future strategies that aim to improve outcomes and enhance user satisfaction. The analysis of perceived barriers, guided by the COM-B model, aims to offer valuable insights into the factors affecting participants' capability, motivation, and opportunity to engage meaningfully with the SP intervention.

Despite the growing interest in SP as an innovative approach to producing health outcomes, the difficulty in evaluating its effectiveness through conventional clinical outcomes is pointed out, particularly when relying exclusively on quantitative methods (25, 26). The subjective nature of the experiences lived by people benefiting from SP tends to be more easily captured by qualitative studies, which have shown greater sensitivity to highlighting the impacts of SP (27).

Therefore, this qualitative study aims to explore the acceptability of the REDE D+ program from the perspective of the person living with T2DM, with the following specific objectives:

- To assess the acceptability and satisfaction with the REDE D+ program among the person with T2DM.
- To explore barriers and facilitators to the acceptability and implementation of the REDE D+ program from the perspectives of people living with T2DM.

The findings may offer valuable insights into redefining SP and guiding its implementation in future large-scale applications.

## Methods

This qualitative study is a part of a broader study entitled “*Fundamental Care for the person with T2DM: Feasibility of a Complex SP Intervention,”* registered under number 5078/CES/2022, and received approval from the ARSLVT Health Ethics Committee (Portugal).

It followed a sequential mixed-methods design, in four main stages: a first stage consisting of a systematic literature review (28); followed by a second stage, a descriptive cross-sectional study that allowed the assessment of needs among the people living with T2DM, the validation of a self-management questionnaire on T2DM to Portuguese, and the exploration of the barriers that T2DM person encounter in their self-care (18–20); a third stage, which allowed the establishment of consensus to refine the SP model and the intervention components (21); and, finally, a fourth stage where a pilot study was developed to assess feasibility and where this study fits into the assessment of the acceptability of the SP complex intervention in promoting self-care and health literacy in T2DM. This qualitative study was defined *a priori* as the final phase of the mixed methods design, with the aim of exploring the narratives and perceptions of the person after the completion of the pilot study.

### Study design/setting

This study adopted a qualitative design to assess the intervention acceptability, over a period of 10 weeks between September and October 2024 took place after the SP intervention program (REDE D+) end. The 18 people with T2DM who participated in the REDE D+ program were randomly divided into two groups of nine participants. According to the literature, focus groups should not exceed 12 participants, as recommended, to create a space for sharing and discussion, and allow everyone to participate (29). The two focus groups were conducted in a room at a primary health care unit in the Lisbon metropolitan area.

### Participants/recruitment

The 18 participants with T2DM, who attended the REDE D+ program for 12 weeks, were invited by telephone and face-to-face to participate in the acceptability analysis. All participants signed an informed consent form to participate in the qualitative study.

### Eligibility/exclusion criteria

All the people with T2DM who enrolled in the REDE D+ program during the 12 weeks were considered eligible.

### Sample size and data adequacy

The sample size was defined based on the participation of people with T2DM in the pilot study, following the principle of informative power, which states that the higher the quality and specificity of the content narrated by the participant, the less need there is for a large sample to ensure the robustness of the study (30). Thus, the sample of 18 participants was considered to have high informative power due to: a) the good specificity of the group (people with T2DM who experienced the intervention); b) the dense and rich dialogue generated in the discussions of the two focus groups; c) the use of a pre-established theoretical model, the COM-B model, which guided the exploration of the barriers encountered during the implementation of the intervention. This approach was aligned with the principles defined by *Reflective Thematic Analysis*, seeking to focus on obtaining conceptual depth and exploring the analytical richness that the narratives offered, allowing for interpretive sufficiency and adequacy of the data (31, 32).

### Reflexivity and the researcher's position

The reflective stance adopted throughout the study allowed the researcher's subjectivity to be transformed into an analytical tool (33), while maintaining awareness of the potential bias that this can bring to qualitative analysis. To ensure the trustworthiness of the data, it was it was guaranteed that interpretations emerged from the narratives of the person with T2DM and not from the researchers' own prior assumptions (34).

The team was composed of different professionals with different professional backgrounds in the areas of chronic disease management, community health, and public health. It is also recognized that one of the researchers had a prior connection with the participants due to their involvement in the pilot study, having played the role of mediator (social prescriber) between the primary healthcare context and the community. Although this clinical proximity could induce a courtesy bias, this methodological option was maintained because it could foster a climate of trust and security in the person, considered a contribution to obtaining authentic narratives (35). Aware that direct involvement in the study development could lead to a bias toward more favorable results, we sought to minimize the risk of bias by making the interviews anonymous and assigning a code (e.g., Patient 1–18).

### Data collection

Acceptability was assessed through qualitative data collected via focus groups. The use of qualitative methods in determining acceptability, such as focus groups, is recognized as important, as they provide in-depth knowledge about the perspective of the population under study (36). The focus groups followed a semi-structured guide, available in Supplementary material 1. The focus groups lasted 90 min. All the sessions were recorded after the participants provided their informed consent, and verbatim transcripts were generated. The verbatim transcripts of the recorded data were subjected to inductive thematic analysis via WebQDA software^®^ (37).

The verbatim transcripts were treated as interpretative constructions in accordance with *Reflective Thematic Analysis* of Braun and Clarke (38). In order to support clarity and preserve the meaning of the narratives, a simple cleaning of the transcripts was performed, removing sounds without contente such background noise, off-topic interruptions, and filler words that did not contribute to the meaning. At the same time, grammar and punctuation were slightly edited to facilitate reading, while maintaining expressions with analytical value.

### Data analysis

The analysis followed a combination of inductive and deductive elements, through an initially inductive coding process, allowing themes to emerge directly from the narratives, without pre-established theoretical restrictions. The verbatim transcripts of the recorded data were subjected to inductive thematic analysis via WebQDA software^®^ (37). The thematic analysis was guided by Braun and Clarke's six-phase approach, which involves: (1) becoming familiar with the data; (2) generating initial codes; (3) identifying potential themes; (4) reviewing and refining themes; (5) defining and naming the final themes; and (6) writing up the findings (38). The transcribed narratives resulting from the different perspectives of each group of participants were analyzed independently by two members of the research team not involved directly to the participants. A third research team reviewed the analyses and conducted a final analysis. Any interpretative differences encountered were resolved through critical dialogue and reflective discussions listed in the context of the narrative and the study objectives, until unanimous consensus was reached. Using a triangulation process among researchers aimed to minimize individual biases and reinforce the credibility of the conclusions presented.

**Figure 1:**
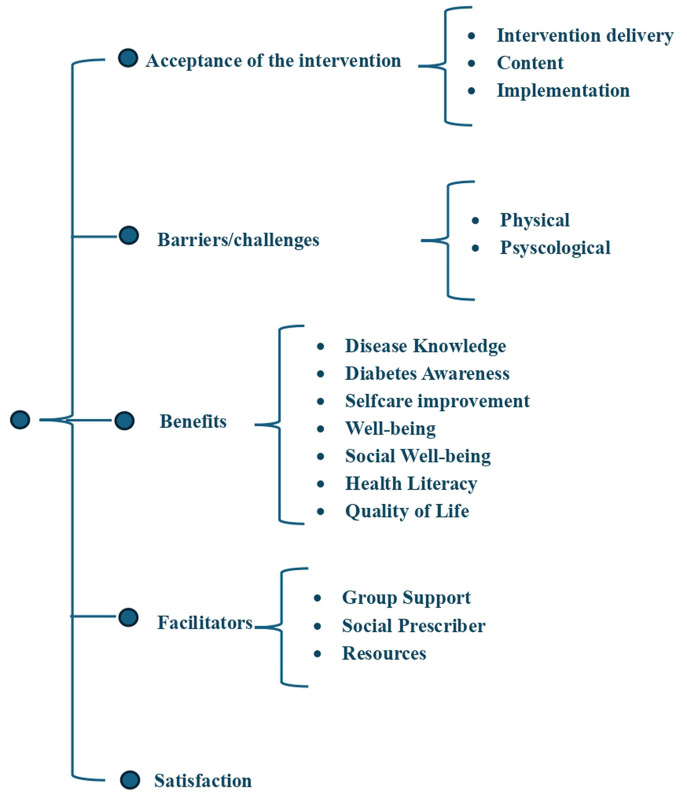
Thematic map of social prescribing acceptability.

This analysis process resulted in the 15 codes organized into five main themes (acceptance of intervention, benefits, barriers/challenges, facilitators, and satisfaction; Figure 1). Although some themes may share a conceptual similarity, each one allows the different meanings to be demonstrated in a distinct and coherent way. The *benefits* were considered to be the positive outcomes perceived by the person (12), while the *facilitators* were intended to reflect the contextual or procedural conditions that enable these same outcomes to be achieved (39). Regarding the SP intervention evaluation, *satisfaction* was related to the subjective and affective evaluation of the quality and usefulness of the intervention received by the person, focusing on the person's experience after contact (40, 41). The theme, *acceptance* was assessed through the lens of the multifaceted concept of acceptability, which sought to reflect the extent to which people who received the intervention considered it appropriate, based on their cognitive or experiential responses (41). These distinctions are intended to ensure that each theme can contribute uniquely to the interpretability of the data.

Subsequently, the theme of *barriers and challenges* was analyzed using the COM-B model (Capability, Opportunity, Motivation) of the BCW. This enabled the identification of key behavioral determinants, capability, opportunity, and motivation, providing a deeper understanding of the factors influencing the implementation of SP in the management of T2DM.

The combination of analyses contributed to the high informative power of the sample (30), as it allowed for both the rigor of a theoretical analysis of barriers and the exploratory depth necessary to capture the richness of positive experiences. By balancing theoretical rigor with exploratory depth, the 18 participants were considered sufficient to achieve interpretative sufficiency, providing a solid mapping of the intervention's implementation.

## Results

### Sample characteristics

The sample of 18 people living with T2DM was characterized by its diversity and heterogeneity. Their ages ranged from 30 to 80 years, with the majority being over 65 years of age (Table 1). The sample included more female participants (72.2%, *n* = 13) and was predominantly retired (71.8%, *n* = 14). Nonetheless, individuals who were employed (11.1%, *n* = 2) or unemployed (11.1, *n* = 2) were also represented, ensuring a broader range of socioeconomic and occupational backgrounds. A similar pattern was observed in terms of educational level, with most participants having completed only primary education (61.1%, *n* = 11). However, the sample also included individuals with different educational profiles, with ≥12 years of schooling (22.3, *n* = 4). Most of the participants had been diagnosed with T2DM for more than six years (72.2%, *n* = 13), and oral therapy (88.9%, *n* = 16), with two combined with the use of insulin (11.1%, *n* = 2).

**Table 1:** Sociodemographic and clinical participants' characteristics.

**Characteristics of participants**	**Frequency *(n)***	**Percent (%)**	**Mean**	**SD (±)**
**Sex**				
Male	5	27.8		
Age	–	–	68.4	± 11.6
**Education**				
Primary education (4 years)	11	61.1	–	–
Lower secondary education (6 Years)	1	5.6	–	–
Upper secondary education (9 Years)	2	11.1	–	–
Postsecondary nontertiary education (12 Years)	3	16.7	–	–
Bachelor's degree	1	5.6	–	–
**Occupation**				
Self-employed	2	11.1	–	–
Retired	14	77.8	–	–
Unemployed	2	11.1	–	–
**Marital status**				
Married	8	44.4	–	–
Divorced	4	22.2	–	–
Widowed	6	33.3	–	–
**Diabetes treatment**				
Oral antidiabetic medication	16	88.9	–	–
Oral antidiabetic medication + Insulin	2	11.1	–	–
**Years diagnosis**				
< 6 years	5	27.8	–	–
≥6 years	14	72.2	–	–

Data are *n* (%) or M (± SD).

### Acceptance of the intervention

#### Intervention delivery, content, and implementation

From the perspective of people living with T2DM as users of the SP intervention (REDE D+ program), it was well accepted. Through narratives, participants conveyed that the information provided about the program was clear and easy to understand from the outset. They perceived a well-coordinated structure and interactions among primary care nurses, the nurse social prescriber, and volunteers/community stakeholders.

“*From the beginning, I felt that with this program, I was charting a course with goals together with my diabetes nurse”* (Patient 18).

“*During this journey, I always felt supported. They communicated with each other, and everyone knew their role. And that gave me a sense of confidence”* (*Patient* 14).

They noted that the intervention structure at each moment was well defined.

“*Everything always seemed to be well organized. We always knew where and what we were going to do. We were always made to feel welcome and easily integrated into the group”* (Patient 2).

The intervention was developed individually and personalized according to each person's needs, to meet their individual goals and requirements.

“*It is different to listen to the doctor or the nurse in the consultation talking about diabetes. It was much better there. I was able to listen to other people, such as me, talking about their doubts, mistakes, worries, and fears. Some of them were just like me”* (*Patient* 3).

“*These activities provided answers to the doubts I had about my illness*” (*Patient 5*).

The duration and frequency of the activities were considered adequate. However, three participants expressed a desire for a greater number of activities during the week, particularly those addressing topics about emotional management and healthy eating, and innovative approaches to food preparation.

“*I think everything was fine, but I would have liked to have had more activities in which we talked about food and our anxieties and worries* (*Patient* 11).

They also highlighted the importance of activities that foster self-exploration, allowing them to reflect on their emotions, personal aspirations, and overall sense of identity.

“*I enjoyed the sessions where we could better understand how what we feel …). It is not easy to start talking; (…) it was hard to expose myself, but with the activities we did as a group, it was very good”* (*Patient* 11).

People with T2DM also considered important how the intervention was delivered, such as the use of messages sent by mobile phone (SMS).

“*Those of us of a certain age need reminders. In addition, messages inform us and remind us that we have something to do. To go to the activity that day or the next day. Or to drink water or walk. It was beneficial.”* (*Patient* 1).

### Benefits

Over 12 weeks, benefits were identified with the intervention development. Better diabetes awareness and its associated problems led to improved knowledge of the different areas of self-care in diabetes: physical activity, diet, medication management, self-monitoring of blood glucose levels with knowledge of values and how to act, and self-monitoring of the feet.

“*It was good to see the change we managed to make with simple things (...). How I now care more about my health and how to manage my diabetes”* (*Patient 7*).

“*Now, when I go to buy shoes, I pay attention to the type of shoes and the shape” (Patient 10)*.

The analyzed narratives showed that the activities improved knowledge about the disease and guided them in simple decision-making.

“*This program has changed the way I see my diabetes. I'm different. Even when I go shopping or to the café, I think about what I'm going to buy to eat. I have made small changes that I think will make a difference” (Patient 18)*.

This recognition and increased awareness prompted people with T2DM to take responsibility, as described.

“*(…) now I recognize that I have a disease that needs care and that I can play an important role” (Patient 15)*.

Moreover, it has led to positive changes in people's behavior and physical and dietary behaviors, with people having the greatest degree of impact. They revealed that they went from being sedentary people, with hardly any physical activity in their daily lives and difficulties in making short journeys, to people who integrated physical activity into their daily lives, improving their mobility and reducing their pain level.

“*I have bettered off my pains, and my hips feel less rusty. With Pilates, I feel better. For a long time, I'd come in at night and find it hard to sleep because of the pains I had” (Patient 4)*.

Behavioral changes focused on the importance of the group's support. The change took place individually via group activities, which allowed them to better understand the disease, improving their self-knowledge through the difficulties they faced, and reflecting on their thoughts and emotions.

“*These activities have brought joy into my life. They were great social moments. Now I have new friends. My life is undoubtedly fuller and better” (Patient 14)*.

Among the reported benefits, we can highlight an improved quality of life and positive feelings that contributed to the participants' wellbeing.

“*I have realized that I do not have to stay closed. (…) this program has done me a lot of good (…). It got me out of the depression I was in. My fears are still there, but I'm better at overcoming them. I have people who have helped me along the way” (Patient 9)*.

They mentioned that community activities fostered social interaction and the creation of new friendships, as well as the development of a support network. This positively impacted their social wellbeing and led to a reduction in isolation.

“*For me, this program was great. Now I meet people when I go out. We arrange walks or short trips (…). I've learned to live again after my husband died. I always took care of others, but now I feel like I'm taking care of myself. (Patient 6)*.

From the reports analyzed, it emerged that the activities enabled the promotion of health literacy with the development of tools by people to define a path, making more consistent, informed decisions in the management of their illness.

“*Now I know more about diabetes and what behaviors I can take a better control; (…), given us the tools (…) to go for information. Now, of course, it is up to me to keep going. Because this is worthless if we do not put it into practice” (Patient 13)*.

#### Difficulties/barriers during the intervention

Primarily, the reported barriers and difficulties were classified as physical and psychological in nature and subsequently analyzed according to the COM-B model. In terms of capability, particularly physical capability, participants identified limitations associated with age-related factors and osteoarticular pain. These physical constraints affected their ability to participate fully in certain activities, particularly those involving movement or walking.

“*My age doesn't help, and my legs are sometimes stubborn*” (*Patient 16*).

Another shared,

“*I was afraid to go hiking because of my knee pain, but I plucked up the courage and went. After doing it, I had some pain, but I went back the following week*” (*Patient 11*).

In terms of psychological capability, two participants experienced initial difficulty understanding what was expected of them using the SP passport. One participant explained,

“*At first, I had some difficulty understanding what I had to write, but after the first face-to-face contact with the nurse (social prescriber) who accompanied me during the program, it was easier*” (*Patient 4*).

Psychological barriers were mostly linked to reflective motivation, encompassing preconceived ideas, challenges in maintaining commitment, and low self-esteem.

“*My age does not help. I thought at 79 that yoga was not for me anymore” (Patient 8). “Now I am different, (...) but I thought I wouldn't be able to do it, that I wasn't capable” (Patient 9)*.

#### Facilitators

Different facilitators were found when the participants' perspectives were analyzed. The importance given to the support group was unanimous.

“*Being in this group was very important for me to realize that other people also have the same problem. With other people's problems and doubts, I have been able to better understand what I can do. The group's help was very important” (Patient 2)*.

The importance of the group, the peer relationship, the resonances between peers, realizing that they are not alone, and the group spirit, were perpetuated even after the project ended.

“*Being in this group was very important for me to realize that other people also have the same problem. With other people's problems and doubts, I have been able to better understand what I can do” (Patient 17);*

“*Joining a group was very important. One person pulls the other along, so it was easier to move things forward. (...) It seemed to me that if I tried to do it alone, I wouldn't get where I got.” (Patient 5)*.

The social prescriber role and the support provided by community stakeholders were emphasized as very important in linking contexts and maintaining a commitment to the objectives set by the person.

“*Having someone such as the nurse (social prescriber) helped us a lot, as it was someone we trusted and who accompanied us throughout this process. It worked like glue that held a patchwork quilt together.” (Patient 8);*

“*The teachers who developed the activities (…) and all the others, such as the social prescriber, played a fundamental role. They helped us to integrate into the group straight away. They made everything much easier; they guided us, they knew how to look at each of us as individuals” (Patient 12)*.

The resources used in the SP Kit, such as the pedometer and the SP passport, were perceived as valuable and motivating tools. The pedometer served as a stimulus for physical activity, encouraging participants to move more and remain active. The SP passport was regarded as a comprehensive resource, offering information on various components of self-care in T2DM, including available community-based activities, a personalized schedule, and a habit-tracking tool.

“*The passport was a part of my day-to-day life on this trip. It allowed me to get to know my disease better, and the activities. I can do in the place where I live, and it made me want to know more. I liked the idea of the QR codes also” (Patient 3)*.

### Satisfaction

Overall, all the participants demonstrated their satisfaction with taking part in the program through their perspectives.

“*It was very good; there was a great return on me. It was a real and important experience. I never thought I would enjoy it so much” (Patient 2)*.

“For me, it was one of the best things that ever happened to me” (*Patient 8*).

## Discussion

This qualitative study reports on the acceptability of the REDE D+ program, a SP intervention, based on the experience of individuals living with type 2 diabetes who participated in the REDE D+ program for 12 weeks. This analysis allows a deeper and more realistic understanding of the mechanisms underlying SP intervention, offering more detailed insights into the perceived benefits, barriers, and behavioral or psychological improvements reported by participants.

The participants' narratives suggested a high level of intervention acceptability, highlighting the importance of complex health strategies that emerge from a deep understanding of the target population's specific needs and local contexts. Notably, despite more than 60% of participants having only 4 years of formal education, most described the information provided during the intervention as clear and accessible. This suggests that the materials and communication strategies were appropriately tailored to the literacy levels of the target population, facilitating understanding and active engagement.

The majority considered the frequency and duration of the activities appropriate; however, three participants suggested increasing the number of sessions focused on nutrition and emotional management. This feedback advises that, in a future large-scale implementation study, the SP intervention could be refined to allow for greater flexibility in the frequency of activities tailored to individual needs, particularly in these two key areas.

The structured design of the program, which fosters a collaborative and interprofessional support network among health professionals, social prescribing nurses, volunteers, and community stakeholders, suggests that it would promote good adherence to the REDE D+ program and a high level of satisfaction among participants. A central feature of this program's structure is its approach based on *Fundamentals of care Framework* (10, 14). An approach centered on the person and their individuality, which is developed according to each person's goals and activities adapted to individual preferences, health conditions, and psychosocial contexts.

Among the most frequently mentioned benefits were increased health literacy, particularly concerning diabetes and its complications. Participants' demonstrated improved understanding of various self-care domains, including physical activity, healthy eating, and self-monitoring. This knowledge was described as essential for making informed, everyday decisions, thereby promoting greater autonomy in disease management (28). This learning process seems to have been accompanied by a growing sense of personal responsibility and awareness about the disease. As participants became more aware of their condition and its implications, they described feeling progressively more empowered to take an active role in their health, aligning with the principles of patient empowerment and self-management (42). Empowering participants to make more informed decisions not only facilitates more efficient use of available resources but also promotes the adoption of preventive practices, directly impacting disease complications (43).

In addition to the knowledge acquisition, narratives suggest behavioral changes, specifically in physical activity and eating habits. Some have transitioned from more sedentary lifestyles to more active routines, resulting in improved mobility, reduced pain, and increased physical wellbeing. These changes were supported by group-based activities, in which peer support played a crucial role, through the sharing of experiences and challenges that fostered a sense of belonging and mutual encouragement, which are recognized enablers of sustained behavior change (44).

Despite the overall positive reception, the participants' narratives also revealed barriers and challenges that influenced their engagement. The use of the COM-B model was fundamental to understanding the mechanisms underlying the acceptance and implementation of the intervention (45), allowing us to identify limitations experienced by individuals. The predominance of retired participants with an average age above 65 suggested a population with greater availability of time, which may have facilitated adherence to the proposed activities. However, this profile may also entail additional challenges related to aging, such as physical or cognitive limitations, which should be considered when adapting intervention strategies. In line with other SP studies (46–48) physical capacity was found to be related to age and osteoarticular pain. We can also reflect on how reduced psychological capacity can negatively influence reflective motivation. One specific barrier to psychological capacity mentioned was the difficulty in understanding and using the SP passport, which caused some frustration among participants. This lack of initial capacity seemed to fuel low self-esteem, negative preconceived beliefs, and maintenance of commitment. From our analysis, we can suggest that these physical limitations may directly affect involvement, increasing the perceived effort required for the person to participate. These internal factors are considered inhibitors of sustained behavioral change, particularly among individuals with long-term chronic conditions. Health professionals and community stakeholders should be aware of their management in relation to the person. Incorporating strategies that promote self-efficacy, emotional resilience, and positive reinforcement becomes essential for long-term support and engagement, as well as for behavior change (49).

The role of the group and the support it provided emerged prominently across participants' shared experiences. This dynamic appears particularly relevant given that a significant portion of the participants lived alone, either widowed or divorced, and the majorities were retired. By fostering community engagement and peer support, the intervention may have partially addressed this gap. Participants with T2DM consistently highlighted the value of peer relationships, the sharing of lived experiences, and the emotional resonance generated through group interaction. These elements contributed not only to a sense of belonging but also to motivation and behavioral reinforcement, underscoring the importance of social connectedness in health-promoting interventions. This collective environment favored the growth of emotional security and motivation, reinforcing the participants' commitment to self-care and lifestyle changes. These findings are consistent with the existing literature on the role of peer support in managing chronic diseases (50, 51).

The role of the social prescribing nurse was also considered fundamental, acting as a bridge between primary care and community resources. This aligns with findings from other studies that highlight the role of social prescribers in enhancing decision-making capacity and self-efficacy in chronic disease management (52–54). Participants appreciated the personalized guidance and continuity provided by this role, which helped to stay focused on individual goals and ensure that the intervention remained relevant to their evolving needs. From another perspective, the involvement of community stakeholders and volunteers was also highlighted, with suggestions for strengthening the support network, creating a cohesive and responsive environment that fostered sustained engagement (55).

It was also suggested that brief messages sent by phone and the SP Kit (namely, the pedometer and passport) were important motivational and co-educational resources for adherence to the intervention and the production of results in self-care and health literacy. The brief messages were perceived as timely reminders and motivational prompts, reinforcing self-care behaviors and maintaining a sense of connection between sessions. The literature supports the effectiveness of personalized short messages sent by mobile phone, especially those based on the BCW framework and incorporating the behavior change techniques, in promoting medication adherence and facilitating sustainable behavior change among people with T2DM (56). This reinforces the importance of incorporating tools in health interventions that support goal setting, self-monitoring, promote motivation and self-efficacy, as well as the development of self-management skills, contributing to improving health literacy and informed decision-making.

### Limitations and strengths

Although the REDE D+ program provided a supportive environment, the findings suggest that future iterations should pay close attention to some of the barriers mentioned, particularly those related to physical capability and motivation. On the other hand, the identified facilitators contributed to the creation of a supportive ecosystem that enabled participants to overcome obstacles, stay motivated, and make meaningful changes in their health behaviors. The integration of peer support, professional guidance, and practical tools proved to be an effective combination in promoting engagement and sustaining SP interventions.

Among the limitations that can be pointed out, include a predominantly older and retired sample belonging to a specific geographical and organizational context, which may in some way limit the results transferability. Although generalization is not the aim of qualitative research, we recognize that experiences with younger adults or with different care contexts may differ. Although the sample was limited to 18 people, this study sought interpretative generalization and depth of understanding of the narrative of those who experienced the intervention. Another potential limitation stems from a predominance of positive reports in the focus groups, which may reflect a social desirability bias, possibly influenced by the moderator's prior connections with the participants. However, to minimize bias, an independent analysis with data triangulation was conducted. This was done to reduce the researcher's subjectivity and ensure the credibility and validity of the data, even though contextual influence cannot be completely excluded.

For future studies, we believe it will be important to include the perspectives of healthcare professionals, particularly nurses, as well as community stakeholders. Actively listening to their experiences can enrich the understanding of the factors that influence the implementation and sustainability of these interventions, contributing to a more comprehensive, collaborative, and context-sensitive approach within primary health care settings.

## Conclusions

The REDE D+ program demonstrated strong acceptability among participants, underscoring its relevance as a complementary strategy in the management of T2DM within primary care. By bridging clinical and community contexts, the intervention provided continuous and integrated support for proactive self-care and health literacy, tailored to individual needs and local resources.

Its multicomponent design, combining personalized care, peer support, and community involvement, was perceived as meaningful and empowering by people living with T2DM, fostering motivation for behavioral change. These findings highlight the potential of SP intervention as an effective and sustainable approach to enhance diabetes care.

Future iterations should consider integrating tailored SP interventions, like the REDE D+ program, into the national healthcare system. This integration should be aligned with the Fundamentals of Care framework, ensuring that healthcare professionals and community stakeholders are actively involved in delivering person-centered, inclusive, and sustainable care for individuals living with T2DM.

## Data Availability

The original contributions presented in the study are included in the article/Supplementary material, further inquiries can be directed to the corresponding author.
