# Supplementary material for: Acceptability of a social prescribing program (REDE D+) for self-care and health literacy among people with type 2 diabetes mellitus—a qualitative study
Source: Front Public Health. 2026 Mar 19;14:1762723. doi: 10.3389/fpubh.2026.1762723 (PMC13044015; doi:10.3389/fpubh.2026.1762723)
Supplement: Supplementary file 1 [file Data_Sheet_1.docx]

**1. Focus Group Roadmap – T2DM patients**

1. How was your experience participating in the Program REDE D+?

2. What did you like about the REDE D+ program?

3. What do you like least about the REDE D+ program?

4. Did you experience any difficulties in developing the REDE D+ program?

5. Considers that the community's activities meet their objectives/needs (knowledge, nutrition, physical activity, medication, self-monitoring).

6. What do you think about the frequency and duration of the activities?

7. What do you think of the SMS messages you received to remind you of activities, day, time, and place, and the reinforcement reminders about self-care?

8. What did you think about the use of the Kit of Social Prescribing (bag, pedometer, and social prescribing passport?

9. What has changed or is changing in your T2DM selfcare behaviour with the REDE D+ Program?

10. To finalize, what did you find most important about your participation in this program?
